# The Correlation between Troponin and Ferritin Serum Levels in the Patients with Major Beta-Thalassemia

**Published:** 2013-06-01

**Authors:** Iraj Shahramian, Motahhare Razzaghian, Abbas Ali Ramazani, Ghasem Ali Ahmadi, Noor Mohammad Noori, Ali Reza Rezaee

**Affiliations:** 1 Department of Pediatrics, Zabol University of Medical Sciences, Zabol, IR Iran; 2 Departement of Epidemiology, Zabol University of Medical Sciences, Zabol, IR Iran; 3 Departement of Pathology, Zabol University of Medical Sciences, Zabol, IR Iran; 4 Pediatric Cardiology, Children and Adolescent Health Research Center, Zahedan University of Medical Sciences, Zahedan, IR Iran; 5 Departement of Biochemistry, Zabol University of Medical Sciences, Zabol, IR Iran

**Keywords:** Major Beta-Thalassemia, Troponin, Ferritin, Cardiac Involvement

## Abstract

**Background:**

Thalassemia is a hereditary hemoglobinopathy whose most common complication is cardiac involvement which ends up in these patients’ death. Since troponin is a sensitive and specific marker for the detection of microinfarct, we studied the relationship between troponin and ferritin serum levels for early diagnosis of cardiac involvement in these patients.

**Materials and Methods:**

This case-control study was performed on 80 patients, including 40 patients with major thalassemia and normal echocardiography and 40 healthy volunteers ranging from 6 months to 16 years old. All the children were examined and the eligible children who were not infected with known heart disease, iron deficiency anemia, kidney disease, diabetes, fever, and systemic diseases were enrolled into the study after obtaining written informed consents from their parents. At 8:00 A.M. before breakfast, 5cc blood was drawn from these children. After collecting the samples, ferritin and troponin serum levels were evaluated using ELISA and electro- kymonolonsense methods, respectively. The gathered data were analyzed through the SPSS statistical software (v. 20) and T-test. Besides, P value<0.05 was considered as statistically significant.

**Results:**

The study results revealed a significant difference between the two groups regarding the mean of the serum levels of troponin (P=0.045) and ferritin (P=0.001). In this study, no significant correlation was observed between serum troponin and ferritin levels and age and BMI in the two groups. Also, no significant relationship was found between serum troponin level and sex (P=0.264).

**Conclusions:**

In microinfarct, troponin increases independent of ferritin; therefore, it can be used for early detection of cardiac involvement in thalassemia patients to determine the sub-clinical effects.

## 1. Background

Major Beta-thalassemia is a hereditary hemoglobinopathy with mutations in the Betaglobin chain that leads to the area hemolysis in these patients (1). Cardiac involvement is the most important and prevalent complication of Beta-thalassemia (2,3). Cardiac involvement in these patients is affected by factors, such as hemocirtus of ferritin (4), destructive effects of free radical oxidation of meiotic membrane and loss of intercellular organelles (5,6), structural changes resulting from chronic anemia due to ineffective erythropoiesis (7), antioxidant presence of 4 allele Apolipo-protein E (8), and microinfarct caused by hypoxia (9).

Ferritin which is a diagnostic marker of iron overload in thalassemia patients (10) increases due to inadequate erythropoiesis, iron overload, transfusion, and increased GI absorption (11). The presence of GDF15 in these patients reduces Hypsydyn (11). Moreover, mutations in the hypophis-hypothalamus axis in JAK STAT pathway enhance iron absorption and are followed by an increase in iron absorption (12). In general, iron deposits in the patients with saturation of reticuloendothelial resources in parenchyma tissues, including the heart. This Iron increase causes free radical formation, enforces the oxidative action on the lipid of cardiomyocyte membranes, and eventually results in damage (13).

T troponin is a delicate filament made of myocyte contractile proteins with 32 amino acids (14) that can be found in released form or in combination with T.I.C. complex (15). In this complex, N-Terminal c.Tn.T segment has a protective role in ischemia and HF. 

"Hypertrophic cardiomyopathy is considered by the occurrence of unexplained left ventricular hypertrophy, which is usually asymmetric and involves the ventricular septum. Molecular genetic studies have identified eleven genes that code proteins of the sarcomere that are associated with the hypertrophic cardiomyopathy" (16,17).

Cardiomyopathy occurs in major thalassemia due to ineffective erythropoiesis and chronic anemia and hypoxia (18). Consequently, these patients are more susceptible to ischemia. Troponin is released during cell damage and due to the loss of myocyte contraction force. After starting onset of irreversible cardiomyocyte damage occurred a similar release of intact cTnI and cTnT and their degradation products due to metabolic inhibition of cardiomyocytes. (9,19). Increased troponin levels serve as a sensitive and specific biomarker of myocardial injury during the early stages and refer to the beginning of a microinfarct (20) although no morphological finding was observed to show the damage in the echocardiography (21).

Because of genetic predisposition, these patients have potential for early involvement; however, due to delay in the development of the symptoms and abnormality in the echocardiography, early diagnosis is impossible during the early processes (22). Therefore, we studied the relationship between troponin and ferritin serum levels for early diagnosis of cardiac involvement in patients with major beta thalassemia.

## 2. Methods

In this case-control study, 40 patients suffering from major thalassemia were diagnosed by hemoglobin electrophoresis. These children who were between 6 months and 16 years old had normal echocardiography and had gone to Amir–Al-Momenin hospital of Zabol to receive packed red blood cell. Also, 40 healthy children who did not have thalassemia, had referred to this center for annual examination, and were age- and sex-matched with the thalassemia patients were enrolled into the study. All the subjects were selected through convenience sampling. 

All the children were examined and the eligible ones who had been treated with no drugs and did not have heart disease, iron deficiency anemia, kidney disease, diabetes, fever, and systemic diseases were enrolled into the study after receiving their parents’ informed consents. 

Five ml blood was drawn from all the children before breakfast at 8 A.M. Then, the samples were centrifuged at 5 °C with a round of 3000 g for 10 minutes. The separated serum was kept in -70°C refrigerator until ferritin and troponin measurements. Finally, consideration to the cold chain, it was transferred to the Biochemistry Lab of Zabol University of Medical Sciences. Then, 250 microns were isolated from the patients' serum by using ELISA kit (USA)., to assess ferritin level. Yet, the other half was evaluated by electrokimonolsence kit to assess troponin level.

In this study, the children over 2 years old were weighed using RASA Mark made ​​in IR Iran by an error factor of 100, while those under 2 years old were weighed by MIKA MARK recumbent weighing scale made in Japane by an error factor of 10 g. In addition, the height of under 2 year old children was measured in the recumbent position by using a flat wooden calibration table, while that of the children above 2 years old was measured in the standing position with a scale ruler. Then, the children's BMI was calculated using the BMI formula; i.e., WT_kg_/HT_m_^2^ (weight in kilograms divided by height to the power of 2).

After all, the data were entered into the SPSS statistical software (v. 20) and analyzed using descriptive-analytical statistics, independent T-test and Correlation Coefficient Pearson tests. Besides, P<0.05 was considered as statistically significant.

## 3. Results

In this study, 80 patients who had referred to Amir-Al-Momenin pediatric hospital, Zabol, IR Iran were divided into a case (children with major beta thalassemia receiving a packed red blood cell) and a control group (healthy children attending the pediatric section for check up) each containing 40 patients. According to the results, 45% and 55% of the study subjects were male and female, respectively and their mean age was 10.23±4.15 years. The mean BMI of the subjects was 16.82±2.29. Besides, the mean of serum troponin levels was 19.22±36.07 and 7.29±3.80 in the patients and controls, respectively.

In this study, a significant difference was found between the case and the control group regarding the troponin serum levels (P=0.045) and the results are shown in Table1. In addition, the mean of serum ferritin level was 687.44±157.96 and 103.63±135.31 in the case and the control group, respectively and the difference was statistically significant (P=0.001) (Table 1). Nevertheless, no significant relationship was found between serum troponin and ferritin level (P=0.371), age (P=0.997) , BMI (P=0.933) and numbers of transfusion (P=0.807) in case group). (Table2). Also, no significant relationship was found between serum troponin and sex (P=0.264) and chelation therapy (P=0.903) (Table3). 

**Table 1.** Comparison of the two Groups Regarding Serum Troponin and Ferritin Levels

Parameter (pg/mL)	mean±SD		*P* value
	Case	Control	
Troponin	19.22±36.07	7.29±3.80	0.045
Ferritin	687.44±157.96	103.63±135.31	0.001

**Table 2.** The Relationship between Serum Troponin Level and Ferritin, Age, BMI, and Transfusion in the Case Group

Parameter	The result of the statistical test
Ferritin	r=0.145
	P=0.371
Age	r=0.001
	P=0.997
BMI	r=-0.014
	P=0.933
Transfusion	r=-0.04
	P=0.807

**Table 3.** The Difference between the Serum Troponin Level and Chelator and Sex

		mean±SD	*P* value
Chelator	Yes (n=23)	19.85±46.90	0.903
	No (n=17)	18.38±16.39	
Sex	M (n=17)	11.58±8.91	0.264
	(n=23) F	24.87±47.53	

## 4. Discussion

In this study which aimed to assess the correlation between troponin and ferritin in thalassemia patients, it was found that regardless of chelatortherapy, number of transfusions, hemoglobin, age, sex, and BMI, troponin had significantly increased in the patients compared to the healthy controls. Ferritin levels were also higher in the patients with thalassemia. However, no significant relationship was observed between troponin and ferritin.

Hessel et al. conducted a study on the release of troponin after the cells infection and reported an increase in troponin level (19). Kaplan S. et al. also performed a study entitled “The chemical markers of myocardium on kids” and showed T Troponin as a special heart biochemical marker in the early stages of myocardial injury (20). In fact, after the apoptosis and the cellular metabolic cession, troponin was released from meiotic which resulted in its increase due to the cellular injury in the serum (19). T Troponin increases even in case of the smallest cellular damage and is considered as a sensitive and specific marker in this regard (20).

The findings of the current study revealed a significant difference between the two groups regarding the increase in ferritin. In the same line, Saraya Ak. et al. conducted a study in order to assess the serum ferritin in thalassemia patients and reported an increase in ferritin (23). John C et al. also reported a ferritin increase in their study of the heart iron overload in transfusion-dependent patients (24). In general, this iron overload is caused by two mechanisms: blood transfusion and inadequate erythropoiesis (11). In thalassemia, GDF15 protein, which is the result of mutations in such patients, works as the inhibitor of peptide-Hepcidin hormone and sends it's reducing signal to the liver. Following Hepcidin reduction, iron absorption from the diet is increased by Ferroportin. Therefore, deficient erythrocyte built in the spleen is trapped, resulting in iron release which eventually leads to an increase in ferritin (11,23,24).

The study results showed no significant relationship between troponin and ferritin. Missow et al. also conducted a study entitled “The markers of heart damage in patients with hemoglobinopathy and hemocirtus resulting from transfusion of high levels of troponin and ferritin” and found no relationships between the two (4). In another study, Koren et al. investigated right ventricular myocardial dysfunction in the patients with major beta thalassemia and reported an increase in ferritin in the thalassemia patients with cardiac involvement (25). Furthermore, John C. Wood et al. studied the impact of iron assessment by MRI and stated that an increase of ferritin would end up in an increase in the risk of cardiac toxicity (13). Iron over load decreases DMT1, a transferrin-bonded transporter to transferrin; i.e., an iron-regulatory mechanism in the body's normal physiological conditions. Under iron overloaded conditions, these mechanisms are lost, extra iron is released, and free radicals are formed. Following the formation of oxygen free radicals, damage occurs in the protein and the lipid of the myocyte membrane. Then, the free radical inactivates the ATPase sodium - potassium pump in the sarcolemma membrane and results in abnormality in the potential conductivity in the heart. After the Lysosome damage, the lysosome enzymes enter the miosit cytoplasm and cause the miosit injury and death (5,6). Atiq M. et al. carried out a research entitled “Cardiac disease in beta-thalassemia major: is it reversible?" and reported that the patients under chelation therapy were faced with cardiac involvement as well as higher ferritin. However, other causes of heart involvement included cardiac iron status, involvement of the myocardial fiber numbers, and structural effects caused by chronic anemia (5). In the present study, the patients were affected by longterm chronic anemia and hemoglobin-deprived hypoxia in order to confront the effects of iron overload. The study showed that ferritin and troponin increased independently.

In the current study, no relationship was found between hemoglobin and troponin levels. Adams et al., in their study on the association between anemia and T troponin, reported a relationship between increased troponin and inadequate erythropoiesis as well as decreased hemoglobin and considered it as a reflection of cardio-myopathy injury (7).

The relationship between bone marrow activity and cardiovascular disorders can be related to red blood cell function in circulation and available oxygen and, hypoxia ,and Subsequent lead to ischemia (7). In fact, due to hemoglobin loss, anemia leads to an imbalance of oxygen supply and demand followed by hypertrophy (9). Meanwhile, there is a vicious cycle between improper erythropoiesis and cardiomyocyte injury. Inappropriate erythropoiesis leads to an increase in the plasma volume in order to compensate the hypoxia resulting from cardiomyopathy (18). On the other hand, hypoxia results in decreased vascular resistance and vessel injuries, especially the blood vessel arteries to the miocits. Therefore, several microinfarcts occur in response to hypoxia (18,26).

In this study, chelator application had no significant relationship with troponin level. Hahalis G et al. performed a study entitled “Right ventricular cardiomyopathy in major thalassemia patients” and stated that despite the use of chelation therapy, the heart involvement was highly prevalent although the prognosis of the patients was improved (21). It is emphasized that cardiac involvement in these patients is not just cause of iron overload and a similar increase in ferritin which can prevent heart trouble through chelation therapy. Moreover, the patients in the current study had no proper chelation therapy.

In this study, the number of transfusions showed no significant association with troponin level. N. O. Kuck et al. in a study on evaluation of cardiac function in major thalassemia patients expressed that myocardial siderosis" could cause cardiac dysfunction and depended on the number of transfusions in these patients (27). Moreover, Leon et al. in a study of early detection of heart function in the patients with major thalassemia and iron overload recognized that the number of transfusion periods was effective on cardiac involvement (28). In another study on physiology and pathophysiology of iron cardiomyopathy in thalassemia, John C. et al. 

 Definite regular transfusion in patients with major thalassemia to stop the destructive effects of ineffective erythropoiesis , marrow expansion and improved patients quality of life . In addition, long-term transfusion leads to chronic anemia and, subsequently, high cardiac output and cardiomyopathy (29).

## 5. Conclusion

According to the current study and the above-mentioned findings, Troponin T can play an important prognostic role in early stages of cardiac damage before conventional echocardiography in the patients with major Beta thalassemia. Thus, it would be possible to measure troponin as a sensitive and unique marker for detecting micro- injuries in their early stages and prevent cardiac dysfunction spread in these patients.

## References

[1] Cao A, Galanello R (2010). Beta-thalassemia.. Genet Med..

[2] Fridlender ZG, Rund D (2004). Myocardial infarction in a patient with beta-thalassemia major: first report.. Am J Hematol..

[3] Noori NM, Eshghi P, Shahramian I (2010). Combined Therapy with Desferal and Deferioprone in Improvement of Heart Function in Thalassemic Patients.. Zahedan Journal of Research in Medical Sciences..

[4] Missov E, Mentzer W, Laprade M, Quill L, Sweeters N, Harmatz P (2001). Cardiac markers of injury in hemoglobinopathy patients with transfusion hemosiderosis.. J Am Coll Cardiol..

[5] Atiq M, Bana M, Ahmed US, Bano S, Yousuf M, Fadoo Z (2006). Cardiac disease in beta-thalassaemia major: Is it reversible?. Singapore Med J..

[6] Piperno A, Taddei MT, Sampietro M, Fargion S, Arosio P, Fiorelli G (1984). Erythrocyte ferritin in thalassemia syndromes.. Acta Haematol..

[7] Adams KF, Jr, Mehra MR, Oren RM, O'Connor CM, Chiong JR, Ghali JK (2010). Prospective evaluation of the association between cardiac troponin T and markers of disturbed erythropoiesis in patients with heart failure.. Am Heart J..

[8] Economou-Petersen E, Aessopos A, Kladi A, Flevari P, Karabatsos F, Fragodimitri C (1998). Apolipoprotein E epsilon4 allele as a genetic risk factor for left ventricular failure in homozygous beta-thalassemia.. Blood..

[9] Thygesen K, Alpert JS, White HD (2007). Universal definition of myocardial infarction.. Eur Heart J..

[10] Gujja P, Rosing DR, Tripodi DJ, Shizukuda Y (2010). Iron overload cardiomyopathy: better understanding of an increasing disorder.. J Am Coll Cardiol..

[11] Trinder D, Fox C, Vautier G, Olynyk JK (2002). Molecular pathogenesis of iron overload.. Gut..

[12] Wang J, Pantopoulos K (2011). Regulation of cellular iron metabolism.. Biochem J..

[13] Wood JC (2011). Impact of iron assessment by MRI.. Hematology Am Soc Hematol Educ Program..

[14] Lipshultz SE, Rifai N, Sallan SE, Lipsitz SR, Dalton V, Sacks DB (1997). Predictive value of cardiac troponin T in pediatric patients at risk for myocardial injury.. Circulation..

[15] Kitagawa M, Sugiyama H, Morinaga H, Inoue T, Takiue K, Kikumoto Y (2011). Serum high-sensitivity cardiac troponin T is a significant biomarker of left-ventricular diastolic dysfunction in subjects with non-diabetic chronic kidney disease.. Nephron Extra..

[16] Kracklauer MP, Feng HZ, Jiang W, Lin JL, Lin JJ, Jin JP (2013). Discontinuous thoracic venous cardiomyocytes and heart exhibit synchronized developmental switch of troponin isoforms.. FEBS J..

[17] Yu ZB, Wei H, Jin JP (2012). Chronic coexistence of two troponin T isoforms in adult transgenic mouse cardiomyocytes decreased contractile kinetics and caused dilatative remodeling.. Am J Physiol Cell Physiol..

[18] Fowler NO, Holmes JC (1971). Ventricular function in anemia.. J Appl Physiol..

[19] Hessel MH, Michielsen EC, Atsma DE, Schalij MJ, van der Valk EJ, Bax WH (2008). Release kinetics of intact and degraded troponin I and T after irreversible cell damage.. Exp Mol Pathol..

[20] Kaplan S (1997). Biochemical markers of myocardial injury in children.. Circulation..

[21] Noori NM, Keshavarz K, Shahriar M (2012). Cardiac and pulmonary dysfunction in asymptomatic beta-thalassanemia major.. Asian Cardiovasc Thorac Ann..

[22] Noori N, Mohamadi M, Keshavarz K, Alavi SM, Mahjoubifard M, Mirmesdagh Y (2013). Comparison of Right and Left Side Heart Functions in Patients with Thalassemia Major, Patients with Thalassemia Intermedia, and Control Group.. Journal of Tehran University Heart Center..

[23] Saraya AK, Kumar R, Choudhry VP, Kailash S, Sehgal AK (1985). A study of serum ferritin in beta thalassemia. Iron deficiency and overload.. Am J Clin Pathol..

[24] Wood JC, Tyszka JM, Carson S, Nelson MD, Coates TD (2004). Myocardial iron loading in transfusion-dependent thalassemia and sickle cell disease.. Blood..

[25] Noori NM, Mehralizadeh S (2010). Echocardiographic evaluation of systolic and diastolic heart function in patients suffering from beta-thalassemia major aged 5-10 years at the Zahedan Research Center for Children and Adolescent Health.. Anadolu Kardiyol Derg..

[26] Clark SJ, Eisenhut M, Sidaras D, Hancock SW, Newland P, Thorburn K (2006). Myocardial injury in infants ventilated on the paediatric intensive care unit: a case control study.. Crit Care..

[27] Kucuk NO, Aras G, Sipahi T, Ibis E, Akar N, Soylu A (1999). Evaluation of cardiac functions in patients with thalassemia major.. Ann Nucl Med..

[28] Noori N, Nejatizadeh A, Rajaei S, Mahjoubifard M (2011). Early diagnosis of cardiac involvement Important in beta thalassemia major.. Iranian J Cardiac surg..

[29] Wood JC, Enriquez C, Ghugre N, Otto-Duessel M, Aguilar M, Nelson MD (2005). Physiology and pathophysiology of iron cardiomyopathy in thalassemia.. Ann N Y Acad Sci..

